# Exercise or physical activity and cognitive function in adults with type 2 diabetes, insulin resistance or impaired glucose tolerance: a systematic review

**DOI:** 10.1186/s11556-018-0190-1

**Published:** 2018-01-22

**Authors:** Ren Ru Zhao, Anthony J. O’Sullivan, Maria A. Fiatarone Singh

**Affiliations:** 1 Exercise, Health, and Performance Research Group, Faculty of Health Sciences, University of Sydney, Lidcombe, NSW 2141 Australia; 2 Hebrew SeniorLife and Jean Mayer USDA Human Nutrition Center on Aging at Tufts University, Boston, MA USA; 3 St George and Sutherland Clinical School, UNSW Medicine, Sydney, NSW Australia; 4 Clinical Rehabilitation Centre, University of Longyan, Longyan, Fujian Province 364012 China

**Keywords:** Diabetes, Exercise/physical activity, Cognition

## Abstract

**Background:**

Diabetes is an important risk factor for cognitive impairment. Although some studies suggest that physical exercise can minimize age-related cognitive declines or improve brain morphology or function, benefits in diabetes or impaired glucose tolerance are unclear. Therefore, our aim was to evaluate the efficacy of exercise or physical activity on cognition in adults with type 2 diabetes, insulin resistance or impaired glucose tolerance.

**Methods:**

An electronic search for studies published from the earliest record until February 2017 was conducted using Medline, EMBASE, SPORTDiscus, CINAHL, and PsycINFO. Any experimental or observational study designs were included, as long as they were conducted in individuals of any age with type 2 diabetes, insulin resistance or impaired glucose tolerance, and they directly examined exercise/physical activity effects on cognitive outcomes or the relationship between changes in cognition and changes in either insulin resistance and glucose homeostasis. Study quality was assessed using the PEDro scale; data on participant and intervention characteristics and outcomes were extracted.

**Results:**

Six studies enrolling 2289 participants met the eligibility criteria. Quality was modest and effect sizes variable and mostly small or negligible. Overall, four of the six studies (67%) reported significant benefits of greater exercise/physical activity participation for some aspects of cognition, but only 26% of cognitive outcomes were significant across all trials. Clinical improvements in insulin resistance/glucose homeostasis were related to improvements in cognitive function in three studies. Overall results were inconsistent, with benefits varying across exercise types and cognitive domains.

**Conclusions:**

Literature does not provide evidence that physical activity or exercise interventions contribute to a better cognitive function in patients with type 2 diabetes or impaired glucose tolerance. Large-scale, long-term, robust randomized controlled trials are required to determine if exercise improves cognition in this high-risk cohort, and to investigate putative mechanistic links between cognition, body composition, metabolism, and inflammation in diabetes and related metabolic syndromes.

## Background

Diabetes is projected to affect 435 million adults by 2030 [1], and as the prevalence rises with age from 12% in people aged 65 to 70 to 15% in those over age 80, older adults will continue to be disproportionately affected [2]. Type 2 diabetes (T2D) accounts for 85% to 90% of patients with diabetes, is a progressive disease manifested by a decline in insulin sensitivity with insulin deficiency that results in sustained hyperglycemia [3], and is the leading cause of cardiovascular disease, kidney disease, vision loss, and neuropathy [4]. Patients with T2D may present with cognitive deficits, associated with reduced performance in multiple domains of cognitive function [5–8]. Higher levels of glycosylated hemoglobin (HbA1c) have been negatively associated with cognitive performance in middle-aged and older individuals [5, 9]. Additionally, in older patients with T2D, cognitive deficits in working memory and attention have been observed in the hyperglycemic state during a glucose clamp [10].

Diet and exercise represent the initial treatment approaches to slow progression of metabolic disturbances associated with pre-diabetes and to assist with pharmacological treatment in established T2D. Increased physical activity has clear beneficial physiological effects for older adults with T2D or glucose intolerance or insulin resistance [11, 12], and more recently has been shown to benefit cognition as well [13–15]. Epidemiological evidence consistently links physical exercise with better cognitive performance [13], lower risk for dementia, and reduced pathological changes in the central nervous system [14–16]. Positive effects of aerobic exercise on cognition have been well documented in animal models and in aging clinical populations [17–19]. There is evidence from cross-sectional and prospective brain imaging studies to suggest that aerobic exercise may reduce brain atrophy in older adults, changes that are most striking for brain regions that support executive control processes and memory [17, 20]. Experimental studies have more recently reported benefits of resistance training on cognitive function in older adults [21–23]. Furthermore, regular physical exercise also has potential therapeutic effects on glucose regulation and cardiovascular health, both of which may threaten cognitive integrity when compromised [22, 24, 25]. Although it has been shown that exercise can enhance cognitive function, most studies have been in healthy older adults, and thus the applicability of these findings to older adults at high risk for cognitive decline is less well defined. Indeed, there are recent reviews of the cognitive benefits of physical activity [26, 27], but there is no systematic review of the effects of exercise on cognitive function in people with T2D, insulin resistance (IR) or impaired glucose tolerance (IGT) to our knowledge. Given the increased risk for dementia posed by T2D, it is important to define the utility of exercise or physical activity for this outcome in this cohort specifically.

Therefore, our objective was to systematically review the literature to identify the relative efficacy of various modes of structured exercise or habitual physical activity level in individuals of any age with T2D or IR or IGT on any measure of cognition, including attention, visual-spatial performance, memory, information processing speed, executive control processes, or global cognitive function. Our secondary aim was to identify the potential mechanisms underlying any cognitive benefits by examining relationships between changes in metabolism, body composition, markers of cerebral pathology including amyloid deposition, and cognitive changes after exposure to chronic exercise or physical activity in this cohort.

## Methods

### Systematic review protocol

A systematic review consistent with the Preferred Reporting Items for Systematic Reviews and Meta-Analyses (PRISMA) was conducted [28]. An electronic search was originally performed in June 2014 and updated in February 2017 using database Medline (1946 - Second week February 2017), CINAHL (1982- Second week February 2017), EMBASE (1974-February 07, 2017), SPORTDiscus (1967- Second week February 2017), and PsycINFO (1967- January week 5 2017). The exercise intervention search terms were: [exercise] [training] [physical] [aerobic] [physical capacity] [aerobic capacity] [physical performance] [physical endurance] [motor activity] [resistance] [weight lifting] [strength] [power training] [strength training] [weight-training] [resistance exercise], combined with “or”. Population terms, combined with “or”, were [diabetes] [insulin] [glucose], Cognitive outcomes terms, combined with “or”, were: [Cognition] [Cognitive] [Memory] [Brain] [Mental] [Neurological] [Neuropsychological]. Intervention, Population, and Cognitive outcome terms were combined with “AND” and then searched in “All Fields” with the limits human and English language. Reference lists of retrieved papers and review articles were hand-searched for any additional papers.

### Eligibility criteria

Studies were selected from the initial search if they met all of the following criteria:

1. Population: Persons diagnosed with T2D or IR or IGT with a valid measure of IR or glucose homeostasis, including fasting glucose or insulin, HbA1c, Homeostatic Model of Assessment-insulin resistance (HOMA-IR) or Homeostatic Model of Assessment 2-insulin resistance (HOMA2-IR), glucose clamp, intravenous glucose tolerance test, oral glucose tolerance test variables or meal tolerance test.
2. Intervention: Physical exercise training consistent with the definition of the American College of Sports Medicine: planned, structured, and repetitive physical activity which has as a final or intermediate objective, the improvement or maintenance of physical fitness [29]. Studies of less than twelve weeks’ duration did not meet criteria for chronic training and were excluded. No limitations were imposed based on modality, dose, or intensity. In experimental studies, the exercise intervention did not need to be fully supervised but must have been prescribed and quantifiable, including self-administered questionnaires or activity monitors [30, 31]. If the study was observational, then it had to report the level of physical activity of the cohort by either objective or subjective measures. The design of the study must have been such that the independent effects of exercise or physical activity could be analyzed. In the case of multiple interventions (e.g., diet + exercise), one group must have been treated with diet/other intervention, another with diet/other intervention + exercise, and both groups must have included individuals with T2D, IGT or IR.
3. Outcomes: Any validated neuropsychological test of cognition reported at baseline and follow-up after exposure to exercise or physical activity. The physiological/metabolic profile was included in the secondary aim of examining relationships between changes in physiological/metabolic profile and changes in cognition, studies must have directly analyzed the effects of changes in IR or other metabolic outcomes on the changes in cognitive function outcomes. Such analyses must have included metabolic, body composition or fitness changes as independent variables using simple or multiple regression models unless the independent effects of changes in these parameters could be determined from the statistical models presented.
4. Control group: For experimental studies, any kind of control group was eligible, including no contact, no treatment, waiting list, attention control, sham exercise, or alternative active treatment. For observational studies, cohorts had to be stratified by level of reported or observed physical activity, with the least active stratum considered the control/low exposure group, and may have included participants who did not have metabolic disorders in the overall study, as long as the subset with the metabolic disease was analyzed separately.
5. Study design: randomized controlled trial (RCT), non-randomized controlled trial (NRCT), uncontrolled trial (UCT), observational study.
6. Full-length article published in a peer-reviewed English language journal.

### Data extraction and quality assessment

One author (RRZ) conducted the search and extracted all data. After eliminating duplicates, all papers identified by the search strategy were screened by the author, first by title and then by the abstract. Two authors (RRZ and AOS) determined final eligibility by reading the full text of potentially relevant studies. Quality assessment of eligible trials was independently rated on the quality rating Physiotherapy Evidence Database scale (PEDro) [32]. An additional item (“Exercise supervised: yes/no”) was included to identify this important component of exercise, providing a final possible score of 12. Any discrepancies were resolved by consensus, or by a third author (MFS) when necessary. Baseline and follow-up data were extracted, and cohort characteristics, intervention (training type, delivery, volume, intensity and duration), and outcome measures were reported along with relationships and statistical significance levels. Authors were contacted for missing data whenever possible.

### Data analysis

A quantitative meta-analysis of studies was not carried out due to the heterogeneity of exercise interventions, outcomes assessed and measurement tools used and the paucity of studies identified. The systematic review compared exercise with no exercise or strata of physical activity at the end of the intervention/observation period. Measurements are presented as mean +/− standard deviation (SD) and significance was set at *p* < 0.05 unless otherwise indicated, as original study papers reported. Relative effect size (ES) (mean change Treatment – mean change Control) ÷ Pooled baseline SD was calculated for controlled trials where possible [33]. Effect sizes were interpreted according to the method of Cohen as: ‘trivial’ (≤ 0.20), ‘small’ (≥ 0.20 to <0.50), ‘moderate’ (≥ 0.50 to <0.80) and ‘large’ (≥ 0.80) [34].

## Results

### Studies retrieved

**Fig. 1:**
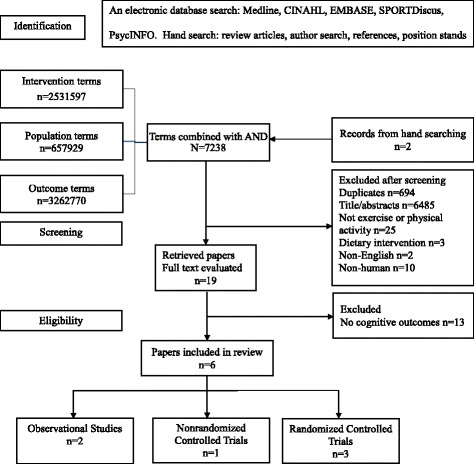
Flowchart of papers identified from search strategy

Figure 1 displays the detailed results of the search process at each step. The combined search identified 7238 potentially eligible studies while hand searching identified a further two studies; this was reduced to 19 after reviewing titles and abstracts, which were reviewed in full to determine suitability. Six studies [35–40] met criteria for this review. This included three RCTs [35, 38, 40], one NRCT [39], one prospective cohort study (which also included a cross-sectional analysis) [37] and one cross-sectional study [36].

### Study quality assessment

An evaluation of the study quality checklist items based on a modified PEDro is summarized in Table 1. Overall, the quality of the three RCTs [35, 38, 40] included in this review was moderate, receiving scores of 7 or 9 out of 12. Common limitations in quality were: lack of blinded assessors, lack of therapists blinded to hypotheses, the absence of intention-to-treat analysis, and insufficient available information regarding baseline matching between groups in all trials [35, 38, 40]. As expected, the NRCT [39], prospective [37], and cross-sectional [36, 37] studies were of a lower standard on the PEDro scale [36, 37, 39], with an average scoring 4/12 (range 4 to 6) [36, 37, 39].

**Table 1:** Study quality of included trials according to a modified PEDro scale

Criterion	Watson et al. 2006 [35]	Colberg et al. 2008 [36]	Devore et al. 2009 [37]		Baker et al. 2010 [38]	Yanagawa et al. 2011 [39]	Lehtisalo et al. 2016 [40]
Study Design	RCT	CS	CS	LS	RCT	NRCT	RCT
Eligibility Criteria	Y	Y	Y	Y	Y	Y	Y
Random allocation	Y	N/A	N/A	N/A	Y	N	Y
Concealed allocation	N	N/A	N/A	N/A	N	N	N
Group baseline similarity	Y	N	N	N	Y	Y	Y
Blinding of subjects	N	N/A	N/A	N/A	N	N	N
Blinding of therapists	N	N/A	N/A	N/A	Y	N	N
Blinding of assessors	N	N/A	N/A	N/A	N	N	N
Measures of one key outcome in at least 85% of participants	Y	Y	Y	Y	Y	Y	Y
At least one key outcomes analyzed with ‘intention to treat’	N	N	N	N	Y	N	N
Point measures, and measures of variability for at least one key outcome	Y	Y	Y	Y	Y	Y	Y
Between group statistical comparisons reported for at least one key outcome	Y	Y	Y	Y	Y	Y	Y
Full supervision of exercise intervention	Y	N	N	N	Y	Y	Y
Final score (/ 12)	7	4	4	4	9	6	7

Points are only awarded when a criterion is clearly satisfied within a modified PEDro Scale. PEDro Scale Physiotherapy Evidence Database Scale, CS cross-sectional study, PS prospective study, RCT randomized controlled trial, NRCT non-randomized controlled trial, N/A, not applicable, Y = 1 N or N/A = 0

### Study design and participant characteristics

A summary of participant and study characteristics can be found in Table 2. Total of 2289 participants were enrolled across 6 studies, ranging from 16 to 1550 participants, with median 71. Pooled sample size amongst RCTs was 578 (398 in exercise intervention arms, ranging from 15 to 364) [35, 38, 40], while 16 participants were enrolled in 1 NRCT [39]. The average age of cohorts across all studies was 64 years, ranging from 56 to 74 years. Sex breakdown was provided in 5/6 study cohorts. Overall, one study included women only [37], while another one did not report sex [35]. The remaining 4 studies [36, 37, 39, 40] were comprised of mixed cohorts.

**Table 2:** Participant characteristics of exercise on cognition in individuals with T2D or IGT

Citation	Inclusion criteria	Condition (N)	Age, years (SD)	% Female	Medications
Watson et al. 2006 [35]	Glucose tolerance criteria for prediabetes 7.8 mmol/L ≥ 2-h glucose <11.1 mmol/L) Normal cognitive assessment	Exercise group: exercise plus AHAS2ED (15) BMI: 24.7 ± 3.2 kg/m^2^ Control group: AHAS1ED (13) BMI:26.7 ± 4.3 kg/m^2^	58.0 ± 9.7 60.6 ± 9.0	Not reported	Not reported
Colberg et al. 2008 [36]	American Diabetes Association criteria for the diagnosis of type 2 diabetes. Cutoff point for MMSE score > 24 and SLUMS <20 for high school educated, <15 for less educated	Diabetes group (74) HbA1c, % 6.6 ± 0.2 Control group (71) HbA1c, % 5.2 ± 0.0	55.5 ± 1.0 57.5 ± 1.3	66.2	Not reported
Devore et al. 2009 [37]	American Diabetes Association criteria for diagnosis of type 2 diabetes Diabetes duration: 1–15 year Telephone Interview for Cognitive Status (modified forms of the Mini-Mental State Examination	High physical activity group (512) BMI (43%) ≥ 30 kg/m^2^ Moderate physical activity group (520) BMI (33%) ≥ 30 kg/m^2^ Low physical activity group (518) BMI (32%) ≥ 30 kg/m^2^ No control group	74.0 ± 2.3 74.0 ± 2.4 74.0 ± 2.3	100	Insulin therapy Oral hypoglycemia Anti-hypertension/ anti-hypoglycemia
Baker et al. 2010 [38]	Glucose tolerance criteria for prediabetes (7.8 mmol/L ≥ 2-h glucose <11.1 mmol/L) and for diagnosed type 2 diabetes (2-h glucose ≥1.1 mmol/L) Normal cognitive assessment	Exercise group: aerobic exercise (19), 2-h OGTT glucose levels:184 ± 46 mg/dL BMI:30.6 ± 3.9 kg/m^2^ Control group: stretching (9) 2-h OGTT glucose levels: 163 ± 31.8 mg/dL BMI: 30.1 ± 7.3 kg/m^2^	71.0 ± 7.5 66.0 ± 6.0	64.3	Anti-hypertension
Yanagawa et al. 2011 [39]	World Health Organization criteria for diagnosis of type 2 diabetes euglycemic clamp) Normal cognitive assessment	Exercise group (9) HbA1c, % 7.29 ± 0.56 BMI: 23.31 ± 2.80 kg/m^2^ Control group: walking (7) HbA1c, % 7.14 ± 0.61 BMI: 22.31 ± 2.03 kg/m^2^	71.6 ± 3.8 70.1 ± 3.7	31.2	Not reported
Lehtisalo et al. 2016 [40]	Glucose tolerance criteria for prediabetes 7.8 mmol/L ≥ 2-h glucose <11.1 mmol/L Normal cognitive assessment	Exercise group: Mixing exercise (364) Control group: Health advice (158)	55.1 ± 6.8 55.3 ± 7.8	67.1	Not reported

*AHAS1ED* American Heart Association Step 1 Eucaloric Diet, *AHAS2ED* American Heart Association Step 2; Eucaloric Diet, *MMSE* Mini-mental State Examination, *SLUMS* Saint Louis University Mental Status Examination, *OGTT* oral glucose tolerance test, *HbA1c* glycosylated hemoglobin, *BMI* Body mass index, *SD* Standard deviation

### Measures of glucose homeostasis and insulin resistance

One study utilized fasting glucose ≥7 mmol/L, or insulin resistance 100 to 125 mg/dL for diagnosis of type 2 diabetes [36]. The hyperinsulinemic euglycemic clamp (the gold standard measurement for IR) was only used in two studies [38, 39], while only two studies used an oral glucose tolerance test to assess glucose tolerance and insulin sensitivity [35, 40]. One study failed to report any measure of insulin or glucose metabolism [37].

### Measures of body composition

Body composition was reported in all but one cross-sectional study [36]. One study used computerized tomography to measure intra-abdominal fat [35] and two studies assessed percent body fat [38, 39]. The remaining two studies reported that participants were overweight or obese, with body mass index (BMI) mean of ≥25 kg/m [37, 40].

### Neuropsychological assessment

Cognitive function was assessed by neuro-psychological examination [35, 38–40]. One study measured cognitive status with the Mini-mental State Exam (MMSE) [36], with a cutoff point for MMSE scores > 24 and Saint Louis University Mental Status (SLUMS) scores <20 for high school educated, <15 for less education inclusive. One study used modified forms of the MMSE [37], and although MMSE scores were inclusionary criteria for the study, the actual scores (global cognitive scores) were not provided [37].

### Intervention characteristics

Tables 3 provides an overview of the exercise interventions among the 4 experimental studies. For the cross-sectional and prospective studies, physical activity exposure was assessed by self-report questionnaires (Table 4).

**Table 3:** Intervention and control characteristics of exercise on cognition in individuals with T2D or IGT

Citation	Intervention modality	Intensity	Volume (minutes)	Frequency (days/week)	Duration (weeks)	Control condition
Watson et al. 2006 [35]	Aerobic exercise plus Diet control Supervised	50% of HRR over 12 weeks increasing to 70% of HRR from 13 to 26 weeks	60	3	52	Stretching plus diet
Baker et al. 2010 [38]	Aerobic exercise, treadmill Supervised	Workload gradually increased to 75–85% of HRR over 1–6 weeks	45–60	4	26	Stretching or balance
Yanagawa et al. 2011 [39]	Aerobic exercise (JOBA) Supervised	55–69% heart rate reserve, or 40–59% of VO_2peak_	45	4	12	Usual level of activity
Lehtisalo et al. 2016 [40]	Brisk walking, skiing, jogging, swimming, bicycling, gymnastics, resistance training, and ball games Supervised	Moderate-vigorous	30	7	156	No any exercise health advice

*JOBA* horseback riding simulation equipment, *VO*_*2peak*_ peak oxygen uptake, *HRR* heart rate reserve, *T2D* type 2 diabetes, *IGT* impaired glucose tolerance

**Table 4:** Intervention and control characteristics of physical activity on cognition in individuals with T2D or IGT

Citation	Physical activity assessment method	Physical activity category ^a^		
		Low active	Moderate active	High active
Colberg et al. 2008 [36]	Modified (diabetes) a version of the HAPAQ (One-year recall)	**Regular exercise, % 55.4** ^b^ Days of exercise/week 2.2 ± 0.3 City blocks walked/day1.2 ± 1.5 Stairs climbed/day flights 0.5 ± 0.6 Usual walking pace 2.0 ± 0.1 Usual exertion 0.9 ± 0.2 **Weekday, hours/day** Vigorous activity 0.8 ± 0.2 Moderate activity 3.0 ± 0.3 Light activity 5.5 ± 0.4 Sit activity 7.1 ± 0.5 Sleep/recline activity 7.6 ± 0.2 **Weekend, hours/day** Vigorous activity 1.0 ± 0.2 Moderate activity 3.2 ± 0.3 Light activity 4.5 ± 0.3 Sit activity 7.1 ± 0.4 Sleep/recline activity 8.2 ± 0.2		**Regular exercise, % 71.8** ^b^ Days of exercise/week 2.2 ± 0.3 City blocks walked/day 0.3 ± 2.4 Stairs climbed/day flights 0.6 ± 0.8 Usual walking pace 0.5 ± 0.1 Usual exertion 4.3 ± 0.2 **Weekday, hours/day** Vigorous activity 0.8 ± 0.1 Moderate activity 3.2 ± 0.4 Light activity 6.9 ± 0.3 Sit activity 5.4 ± 0.4 Sleep/recline activity 7.6 ± 0.2 **Weekend, hours/day** Vigorous activity 0.2 ± 0.2 Moderate activity 4.2 ± 0.3 Light activity 5.4 ± 0.3 Sit activity 5.3 ± 0.4 Sleep/recline activity 7.9 ± 0.2
Devore et al. 2009 [37]	SAPAQ-CS SAPAQ-PS	3.38 (0.13–6.76)	10.70 (6.77–15.50)	24.39 (15.54–112.23)

^a^Physical activity category is classified according to the HAPAQ and SAPAQ where possible (Devore reported exercise intensity as metabolic equivalents (METS) [37], while the study of Colberg et al. (2008) defined the intensity of aerobic activities according to exertion levels (e.g. usual exertion, usual walking pace, stair climbing) [36]

^b^Regular exercise was defined (using the HAPAQ) as participants engaging in at least 30 min of moderate aerobic exercise 3 times a week for a minimum of 1 year

*SAPAQ* Self-administered Physical Activity Questionnaire, *HAPAQ* Harvard Alumni Physical Activity Questionnaire [31], *CS* cross-sectional study, *PS* prospective study, *T2D* type 2 diabetes, *IGT* impaired glucose tolerance

### Training modality

One trial tested the effect of isolated moderate-intensity aerobic exercise [38], one moderate- and high-intensity mixed training [40], and one prescribed low-intensity horseback riding therapeutic equipment (JOBA) compared to a maintaining usual level of activity (walking) control group [39]. Only one study investigated the effects of a combination of aerobic exercise and dietary restriction compared to a diet restriction plus stretching control group [35]. The single prospective cohort study compared the effects of highest habitual physical activity level to lowest physical activity level [37], while the cross-sectional study, derived from the same cohort, examined the difference between the highest and the lowest physical activity groups [37]. Finally, another cross-sectional compared the cognitive function of regular exercise in a sedentary diabetes group (controls) to exercise in a group with T2D [36].

### Exercise dose and intensity

Exercise volume in experimental studies varied from 30 to 60 min per session, three to seven sessions per week, and duration of interventions ranged from 12 to 156 weeks. Two trials of aerobic exercise used exercise equipment at moderate [35, 38], or low [39] intensities (JOBA), whereas moderate- and high-intensity walking was prescribed in one mixed resistance and other exercise modalities [40]. In the prospective cohort and cross-sectional studies, for classification of habitual physical activity, the period of observation was 1 year [36] and 4.2 years of follow-up [37], respectively. Devore et al. (2009) reported exercise intensity as metabolic equivalents (METs) [37], while Colberg et al. (2008) [36] defined the intensity of aerobic activities according to exertion levels (e.g., usual exertion, usual walking pace, stair climbing).

### Control conditions

Control conditions were highly variable and included diet [35], stretching or balance [38], usual activities [39], or health advice [40]. Three of the experimental studies reported supervision during the control condition [35, 38, 40]. The one prospective and two cross-sectional studies described the control condition as a low level of habitual aerobic exercise/physical activity, which was compared to the higher activity level stratum, all unsupervised [36, 37].

### Outcome measurement

A total of 20 different cognitive outcome measures were administered (average 4/study, range: 2–7). Data from non-significant findings were not always provided [35, 38]. The general cognitive function was measured using MMSE, SLUMS, Telephone Interview for Cognitive Status (TICS), or overall global scores [36, 37, 39, 40]. The majority of studies administered standardized neuropsychological tests with attention/executive function the most frequently measured domain [35, 38, 39], followed by memory [35, 37–39] information processing [39, 40], and global cognitive function [36, 37, 39]. Only four trials had a longitudinal follow-up after intervention at 12 weeks [39], 26 weeks [38], 52 weeks [35], or 36 months [40]. Supervision was removed during the maintenance phase in one study and participants were advised to continue exercise at home for the final 6 months [35]. The one prospective cohort study had follow-up over 4.2 years [37].

Four of the 6 studies (67%) reported significant benefits for at least one outcome [35–38]. However, only 26% of cognitive outcomes were significant across all trials. Exercise was reported to result in improved global cognition [36, 37], executive function [38], and memory [39] in these 4 studies, which included 2 interventional and 2 observational studies.

### Executive function/attention

#### Aerobic exercise vs. non-exercise controls

A summary of the results can be found in Table 5. Only one [38] out of four [35, 38–40] studies reported a significantly greater improvement in three of five executive function tests in aerobic training compared to controls, with small effect size (ES) [(Trail Making Test B ES = 0.36, *p* = 0.04), (Task Switching ES = 0.39, *p* = 0.03), and Stroop Color-Word Interference ES = 0.38, p = 0.04)], and trends for improvement in Verbal Fluency (ES = 0.26, *p* = 0.11) and Self-Ordered Pointing Test (ES = 0.29, *p* = 0.10) over 6 months. [38] However, analysis of executive function showed no difference in a trial of low intensity JOBA exercise vs. controls [(Trail Making Test A ES: 0.46; 95% CI: -0.54, 1.46), (Trail Making Test B ES: 0.60; 95% CI: -0.41, 0.41), (Stroop ES: 0.14; 95% CI: -0.84, 1.13), (Wechsler Adult Intelligence Scale Revised-Digit Symbol test ES: 0.38; 95% CI: -0.62, 1.38)] [39]. Similarly, there was no significant effect of exercise on executive function in two other aerobic exercise trials [35, 40].

**Table 5:** Effect of exercise interventions on cognition

Citation	Outcome measurement		Results			Calculation	Statistics
	Domain	Method	Group	Pre-exercise (Mean ± SD)	Post-exercise (Mean ± SD)	Between group Effect size (95%, CIs)	Between group *p*-value
Watson et al. 2006 [35]	Memory Attention/Executive function	SR BVR TMT	AE CO AE CO AE CO	6.30 ± 1.15* 6.25 ± 1.10* Not reported Not reported Not reported Not reported	3.65 ± 1.15* 5.05 ± 1.15* Not reported Not reported Not reported Not reported	1.35 (0.48, 2.22) Unable to be calculated Unable to be calculated	0.01 Not significant Not significant
Baker et al. 2010 [38]	Information processing and executive function Memory	VF TMTB TS SCWI SOPT SR LR	AE CO AE CO AE CO AE CO AE CO AE CO AE CO	Not reported Not reported Not reported Not reported Not reported Not reported Not reported Not reported Not reported Not reported Not reported Not reported Not reported Not reported	Not reported Not reported Not reported Not reported Not reported Not reported Not reported Not reported Not reported Not reported Not reported Not reported Not reported Not reported	Unable to be calculated Unable to be calculated Unable to be calculated Unable to be calculated Unable to be calculated Unable to be calculated Unable to be calculated	0.11 0.04 0.03 0.04 0.10 Not significant Not significant
Yanagawa et al. 2011 [39]	Memory Information processing and executive function Global cognitive function	WLIM WLDM WAIS-DST TMTA Stroop TMTB MMSE	AE CO AE CO AE CO AE CO AE CO AE CO AE CO	Not reported Not reported Not reported Not reported Not reported Not reported Not reported Not reported Not reported Not reported Not reported Not reported Not reported Not reported	Not reported Not reported Not reported Not reported Not reported Not reported Not reported Not reported Not reported Not reported Not reported Not reported Not reported Not reported	0.59 (−0.42, 0.60) 0.08 (−0.90, 1.07) 0.38 (−0.62, 1.38) 0.46 (−0.54, 1.46) 0.14 (−0.84, 1.13) 0.60 (−0.41, 0.41) 0.02 (−0.97, 1.00)	0.31 1.00 0.79 0.53 0.27 0.27 0.96
Lehtisalo et al. 2016 [40]	Overall cognitive performance Executive function	CERAD TMTB	AE CO AE CO	8.3 ± 9 Not reported 48.6 ± 9.6 Not reported	Unable to be calculated Unable to be calculated	Unable to be calculated Unable to be calculated	Not significant Not significant

Data with an asterisk were estimated from graphic values provided using the Matlab software. Between-group and/or intra-group effect sizes (ESs) were calculated for both groups. Between group effect size = (Change in Treatment – Change in Control) / Pooled baseline SD [33]. Intra-group effect size = (post-exercise - pre-exercise) / pooled baseline SD [34]. Data are presented as mean ± SD and effect size and 95% confidence intervals (CIs), adjusted for age, BMI and years of education

*SD* standard deviation, *ES* effect size, *CIs* confidence intervals, *AE* aerobic exercise, *CO* control, *SR* Story Recall (Story Recall (immediate recall and delayed recall), *BVR* Benton Visual Retention, *TMT* Trail Making Test, *SIT* Stroop Interface Test, *VF* Verbal Fluency, *TMTA* Trail Making Test A, *TMTB* Trail Making Test B, *TS* Task Switching, *SCWI* Stroop Color-Word Interference, *SOPT* Self-Ordered Pointing Test, *LR* Learning Recall, *WLIM* Word List Immediate Memory a subtest of the Alzheimer’s disease Assessment Scale, *WLDM* Word List Delayed Memory a subtest of the Alzheimer’s disease Assessment Scale, *DST* Digit Symbol Test a subtest of the Wechsler Adult Intelligence Scale-Revised, *MMSE* Mini-Mental State Examination (maximal score = 0–30), *CERAD* Consortium to Establish a Registry for Alzheimer’s Disease

The Finnish CERAD Battery is composed of 1) Verbal fluency (animals) 2) Modified Boston Naming test (15 words) 3) Mini-Mental State Exam 4) Word List Memory (ten words, three trials) 5) Constructional praxis 6) Word List Recall (delayed recall of the ten words) 7) Word List Recognition (recognition of the ten words out of 20 words) 8) Constructional praxis recall 9) Clock drawing

### Memory

#### Aerobic exercise vs. non-exercise or aerobic exercise and diet restriction vs. diet restriction or high physical activity vs. low physical activity

A summary of the results can be found in Table 5. Four studies [35, 37–39] examined the effects on memory and nonsignificant results were reported for the majority of outcomes. Aerobic training combined with diet control compared to a diet control group produced significant large effects on delayed memory (Story Recall Test ES: -1.26; 95% CI: -1.83, −0.70), but nonsignificant results on overall memory domain [35]. Similarly, nonsignificant results were found in studies of low intensity JOBA exercise [39] and moderate intensity treadmill exercise [38]. The cross-sectional analysis failed to find positive effects of high physical activity compared to low physical activity levels [37], and in longitudinal analyses from this same cohort, there was no relationship between physical activity level and memory in this prospective cohort study at the 4.2-year follow-up [37]. Thus, aerobic training and diet compared to diet only control has been shown to improve one memory test in one trial, and no data are available on direct comparisons of the efficacy of different exercise modalities for these outcomes.

### Information processing speed

#### Aerobic exercise vs. non-exercise controls

Results are reported in Table 5. Few data on information processing speed were available, as this was reported after aerobic training in 2 only trials [39, 40]. No significant improvements were shown compared to non-exercise controls.

### Global cognitive function

#### Aerobic exercise vs. non-exercise or higher physical activity vs. low physical activity

As reported in Table 6, aerobic exercise or high physical activity did not significantly improve any global cognitive outcome in a prospective study [37], one RCT [40], and one NRCT [39]. Two cross-sectional analyses reported significantly better global cognition in those with higher physical activity levels however [36, 37]. Similarly, sedentary time showed an inverse relationship with a global cognitive function on the SLUMS test which was used in the study of Colberg et al. [36].

**Table 6:** Effect of exercise /physical activity on cognition

Citation	Assessment time point	Outcome measurements		Results			Statistics
		Domain	Method	Group	Physical activity levels	Between group mean difference (95%, CIs)	*p*-value
Colberg et al. 2008 [36]	Cross section study	Global cognitive function	MMSE SLUMS	AE CO AE CO AE CO	weekday light activity: 5.5 ± 0.4 weekday light activity: 6.9 ± 0.3 weekday moderate activity: 3.0 ± 0.3 weekday moderate activity: 3.2 ± 0.4 weekday light activity: 5.5 ± 0.4 weekday light activity: 6.9 ± 0.3	Not reported Not reported Not reported	0.04 <0.01 0.04
Devore et al. 2009 [37]	Cross section study	General cognitive function Memory	TICS score Global cognitive Score Immediate and delayed recalls	LPA MPA HPA LPA MPA HPA LPA MPA HPA	3.38 (0.13–6.76) 10.70 (6.77–15.50) 24.39(15.54–112.23) 3.38 (0.13–6.76) 10.70 (6.77–15.50) 24.39(15.54–112.23) 3.38 (0.13–6.76) 10.70 (6.77–15.50) 24.39(15.54–112.23)	0.37 (0.02, 0.72) 0.07 (0.01, 0.15) 0.06 (0.03, 0.1)	0.03 (HPA versus LPA) 0.06 (HPA versus LPA) 0.20 (HPA versus LPA)
Devore et al. 2009 [37]	Prospective study	General cognitive function Memory	TICS score Global cognitive Score Immediate and delayed recalls	LPA MPA HPA LPA MPA HPA LPA MPA HPA	3.38 (0.13–6.76) 10.70 (6.77–15.50) 24.39(15.54–112.23) 3.38 (0.13–6.76) 10.70 (6.77–15.50) 24.39(15.54–112.23) 3.38 (0.13–6.76) 10.70 (6.77–15.50) 24.39(15.54–112.23)	0.21 (0.15, 0.57) 0.05 (0.03, 0.12) 0.07 (0.03, 0.16)	0.20 (HPA versus LPA) 0.20 (HPA versus LPA) 0.20 (HPA versus LPA)

Data are presented as mean ± SD and mean difference and 95% confidence intervals (CIs), adjusted for age, education, disability indicators, and others

*SLUMS* Saint Louis University Mental Status exam, *MMSE* Mini-Mental State Examination, *TICS* Telephone Interview for Cognitive Status, *LPA* low physical activity, *MPA* moderate physical activity, *HPA* high physical activity, *AE* regular physical exercise in individuals with diabetes, *CO* regular physical exercise in normal individuals

### Relationship between changes in measures of cognitive function and insulin resistance

Results are shown in Table 7. Improvements in delayed memory were correlated with improvements in glucose infusion rates (*r* = 0.64; *p* = 0.024) during a euglycemic clamp, with a trend for metabolic clearance rate (*r* = 0.575; *p* = 0.051) in those who performed JOBA exercise, as well as in the usual physical activity control group [39]. In another intervention study, improvements in delayed memory were related to greater reductions in 2-h OGTT insulin levels (*r* = −0.52; *p* < 0.05) in the aerobic exercise plus diet group only, while no relationship was seen in the diet and stretching control group [35]. Additionally, global cognitive function in participants reporting higher levels of physical activity was inversely related to HOMA-IR (*r* = −0.19, p = 0.02) and insulin level (*r* = −0.13, *p* = 0.03) in a cross-sectional study [36]. Thus, there is some evidence to suggest that improvements in insulin sensitivity are either potentially contributory to cognitive adaptations or at least markers for other beneficial mechanistic adaptations and are modified proportionally by exercise.

**Table 7:** Relationship between changes in cognition and insulin resistance

Citation	Cognition	Insulin resistance measurement	Time since last exercise bout	Relationship	r	*p*-value	Comments
Watson et al. 2006 [35]	Delayed memory	Two-hour Oral glucose tolerance test insulin level (mmol/L)	26 weeks	Change in delayed memory and change in two-hour Oral glucose tolerance test insulin level (mmol/L)	−0.52	0.047	Exercise group (Aerobic exercise plus American Heart Association Step 2 Eucaloric Diet only
Colberg et al. 2008 [36]	Global cognitive function	Homeostatic Model Assessment-insulin resistance Insulin (IU/mL)	Not reported Not reported	Relationship between HOMA-IR and cognitive function Relationship between insulin level and cognitive function	−0.19 −0.18	0.02 0.03	Diabetes exercise group
Yanagawa et al. 2011 [39]	Delayed memory	Euglycemic Clamp (glucose infusion rate (mL/kg/min) Metabolic clearance rate(mL/kg/min)	12 weeks	Change in word recall and change in glucose infusion rate Change in word recall and change in metabolic clearance rate	0.64 0.575	0.024 0.051	Horseback riding simulation equipment (JOBA) exercise group

### Relationship between changes in measures of cognitive function and glucose homeostasis

A summary of the results can be found in Table 8. Improvements in word recall were related to reductions in HbA1c (*r* = −0.627; *p* = 0.029) after JOBA intervention in one NRCT but were not observed in the control group [39]. Higher fasting blood glucose was directly related to worse executive function (*r* = 0.611; *p* = 0.035) in this same study [39]. However, no such relationships between cognition and glucose changes were reported in those who performed aerobic training or aerobic training plus diet control only, or in the control groups of two other studies [35, 38]. Thus, there is mixed support for improvements in glucose homeostasis as a mechanism or marker for cognitive benefits of exercise in cohorts with metabolic disease.

**Table 8:** Relationship between changes in cognition and glucose homeostasis

Citation	Cognition	Glucose level measurement	Time since last exercise bout	Relationship	r	*P* value	Comments
Yanagawa et al. 2011 [39]	Delayed memory Executive function	HbA1c (%) (glycosylated hemoglobin) Fasting glucose level (mg/dl)	12 weeks 12 weeks	Change in word recall and change in HbA1c Change in word recall and change in metabolic clearance rate	−0.627 0.611	0.029 0.035	Horseback riding simulation equipment (JOBA) exercise group

### The relationship between changes in measures of cognitive function and body composition

A summary of the results can be found in Table 9. Reductions in intra-abdominal fat (IAF) were correlated with improvements in delayed memory within control participants who received diet and stretching, while no significant relationship was observed in those who received aerobic training and dietary intervention [35]. No other studies reported body composition/cognition relationships, precluding generalized conclusions.

**Table 9:** Relationship between changes in cognition and body composition

Citation	Cognition	Body composition measurement	Time since last exercise bout	Relationship	r	*P* value	Comments
Watson et al. 2006 [35]	Delayed memory	Intra-abdominal fat (cm^2^)	26 weeks	Change in delayed memory and change in intra-abdominal fat (IAF)	Not reported −0.62	Not reported 0.024	Exercise group (exercise plus American Heart Association Step 2 Eucaloric Diet) Control group (stretching plus American Heart Association Step 1 Eucaloric Diet)

## Discussion

This systematic review identified only 3 RCTs, one NRCT, one cross-sectional and one prospective cohort study of exercise or physical activity exposure in individuals with T2D or IGT or IR investigating the relationship between exercise/physical activity and cognition. The quality of this very small literature was only moderate, and the majority of studies had insufficient power due to low sample size to detect small effects. Few cognitive outcomes (26%) were significant, thus providing no strong or consistent evidence that aerobic exercise or lifestyle intervention or higher levels of habitual physical activity improve cognition or are associated with less risk of cognitive impairment/decline in individuals with T2D or IGT or IR.

Abnormal glucose tolerance, a characteristic of prediabetes and T2D, has been related to an increased risk of cognitive impairment and dementia compared with healthy peers [41–43]. Regular exercise has potential therapeutic effects on cognitive function compromised by T2D and Alzheimer’s disease (AD) pathology [41–45]. Long term T2D often contributes to numerous harmful consequences for peripheral systems and neurophysiologic and structural changes in the brain that adversely affect cognition [44] and ultimately increase the risk of dementia [46]. Cerebral neuropathological changes are believed to begin in the early stages of diabetes, consistent with deficits in cognitive performance for adults with prediabetes as well [47, 48]. Exercise and lifestyle interventions may improve metabolic health as well as potentially attenuate neuropathology in adults at high risk for developing type 2 diabetes, thus ultimately both preventing type 2 diabetes and preserving cognitive function. However, there are currently no studies comparing the use of exercise for cognitive benefits at various stages of the disease process, and this is an important topic for future study. Theoretically, it would seem intuitive that halting the neuropathological changes in the brain at an early stage might be most beneficial, however, literature reviews in dementia [14] actually show that the effect size of exercise on cognition is *greater* in those with cognitive impairment and/or dementia than it is in older adults with normal cognition. Given that the adverse effects of hyperinsulinemia on neuronal structures and function can be addressed even after a neurotoxic insult has occurred in animal models [49], there would appear to be the rationale for the application of exercise along the entire spectrum of disease from impaired glucose tolerance to established, longstanding T2D.

Prior studies in cohorts without diabetes have mainly focused on aerobic exercise and linked it to improved executive function [19, 50, 51]. In our review, moderate-to-high intensity aerobic exercise was related to improvements in executive function in only 1 [38] of 4 studies [35, 38–40], and additional evidence is needed to confirm this finding. Exercise may protect executive function by reducing brain atrophy [52], However, the failure to observe consistent beneficial effects of exercise on executive function as shown the other 3 trials is notable. The type of exercise prescribed (low intensity and short duration JOBA training) may have attenuated the response in one study [39]. It is likely that small sample size may have resulted in a type II error in the group with T2D or IGT [35, 39]. The other study [40] which reported no significant improvement in executive function after mixed aerobic training had a high dropout rate of 30% which may have precluded benefit. Additionally, this study included participants of younger ages with a relatively high level of cognitive function even at the end of the follow-up study, and thus even longer-term follow-up may have been needed in this early prediabetes stage due to ceiling effects on cognitive testing utilized. Thus, as there is only one positive study [38] investigating executive function and exercise, further investigation is required to confirm these findings. By contrast, no beneficial improvements were seen for information processing speed domain after aerobic exercise in older adults with T2D [39] or IGT [40]. These findings stand in contrast to large beneficial effects reported in two previous studies of aerobic exercise healthy cohorts [53]. Sample sizes in this review may have been too small to detect a significant improvement in information process speed, and thus replication in larger samples is indicated.

Only one study compared memory changes between exercise and diet control vs. diet control, and reported a significant, large benefit of the exercise on one memory outcome [35]. Although additional studies are needed, this finding is consistent with evidence in older adults without metabolic disease which suggests a moderate effect in favor of aerobic exercise combined with diet management [54, 55]. This may be explained by the fact that participants in the diet plus exercise group had greater improvement in metabolic parameters directly relevant to memory than did the diet only group. However, in the observational study of high physical activity vs. low physical activity in older individuals with T2D, this benefit for memory was not seen [37]. It is possible that long-standing diabetes, medication treatment, and/or disability may have attenuated the response in this cohort. Similarly, the current review did not find any consistent benefit of aerobic training compared to controls for memory in the other experimental studies of older adults with T2D [39] or IGT [38]. Memory tasks are supported by structural plasticity in the hippocampus [56] which has been observed after aerobic voluntary wheel running in rodents. In humans, the absence of exercise-induced memory benefits in most trials we reviewed may relate to the type of tests administered or the specific task demands that rely on patterns of structural plasticity in specific brain regions. Modality, dose, intensity, and duration of testing is another possible explanation. However, no consistent pattern has been demonstrated and further study is needed.

Only two cross-sectional studies and no experimental studies reported that high physical activity vs. low physical activity showed global cognitive function improvements, and thus it is not possible to draw conclusions about the efficacy of physical activity for global cognition in this cohort. Evidence in older people without T2D suggests a small effect in favor of high physical activity for global cognition [57]. Regular physical exercise participation may offset many of the increased risk factors for cognitive dysfunction frequently associated with diabetes, such as visceral obesity and hypertension [45]. Aerobic fitness itself is also linked to cognitive decline, and an increase of only 1.0 MET has been linked to a lower rate of global cognitive decline, mild cognitive impairment and AD in older individuals, for example [58]. Moderate-to-vigorous intensity aerobic exercise is the most potent exercise modality for improving aerobic fitness, thus providing another rationale for its inclusion in exercise programs and guidelines for older adults with T2D. However, the analysis of the cross-sectional/observational studies has some limitations: the level of physical activity was self-reported in two studies, (with physical activity category classified according to the Harvard Alumni Physical Activity Questionnaire and Self-administered Physical Activity Questionnaire, whereas Devore [37] reported exercise intensity as metabolic equivalents, and Colberg [36] defined the intensity of aerobic activities according to exertion levels. Thus, actual physical activity/exercise intensity may not have been reliably captured. Although Devore et al. [37] demonstrated that the validity of such questionnaires is adequate, the results should be interpreted with caution. Among three studies which included patients with T2D [37, 39] or IGT [40], Devore [37] showed that subjects of T2D engaging in a higher level of physical activity per week had better global and verbal cognitive outcomes compared to the lower level of physical activity after adjusting age and education level. However, the initial results disappeared after further adjustments for all covariates. Similarly, among the two experimental studies (one RCT and one NRCT) included in this review, the Lehtisalo [40] did not find any improvement in global cognition in the T2DM or IGT patients after training. This cohort had a relatively young group of participants, with a relatively high level of cognitive function even at the end of their follow-up study. Another possibility is that there were also higher dropout rates after the long follow-up. Furthermore, in the NRCT [39], no positive effects of exercise on global cognition were reported in older adults with T2D. The relatively small sample size and low exercise training intensity and short duration may have contributed to the lack of benefit observed.

None of the trials focused on progressive resistance training (PRT). However, much more research is needed about such anabolic exercise due to its clinical relevance, including prevention of incident dementia and T2D in this high-risk cohort. First, reductions in adiposity and increases in muscle mass, which are targeted by PRT, have been specifically associated with improved insulin sensitivity, glucose control, and inflammation [59–62], which are in turn associated with a decreased risk of cognitive decline. Secondly, many older adults with co-morbidities may not tolerate moderate-to-high intensity aerobic exercise but can tolerate high-intensity resistance training. Resistance training may also improve hypertension, dyslipidemia, insulin and glucose regulation, which are important comorbidities of T2D associated with cognitive impairment [25, 63]. Third, PRT results in a range of positive benefits for neurobiological outcomes in animal and human studies, such as increased insulin-like growth factor 1 (IGF-1) [18], increased brain-derived neurotrophic factor [64–66], neurogenesis [66], functional plasticity [21], decreased inflammatory cytokines [67, 68], decreased cortisol response to stressors, and improved cognitive function [64, 69]. Specifically, PRT can increase IGF-1 levels, which may lead to improved neurogenesis and vessel remodeling in the brain [70]. Currently, the American College of Sports Medicine recommends that exercise programs for older adults include both aerobic and nonaerobic physical activities, such as resistance training, balance training, and stretching for optimal general health [29]. The inconsistent efficacy of isolated aerobic training for cognitive outcomes in this review suggests that for optimal cognitive outcomes in those with metabolic disease, such a combined prescription of exercise modalities may also be required, and requires specific investigation.

The small amount of evidence available suggests that better cognitive outcomes on executive function [39], memory [35], and global cognitive function [36] were partially explained by, or associated with, reductions in IR. In this context, it is notable that reduced insulin elevations were associated with better delayed memory in IGT patients after aerobic training and diet control in one study which was included this review, compared to diet alone. Although suggestive, we cannot conclude that the exercise alone would have had this same association. In another study in this review, improvements in HOMA2-IR were related to improvements in memory and executive function, even if no significant change in either measure was observed post-intervention. The intervention delivered during the negative trial was JOBA exercise [39], and it may be that the intervention was not of a sufficient intensity to induce significant improvements in delayed memory, information processing speed, executive function, or global cognitive function, nor provide robust metabolic benefit. However, what the results do suggest is that improvements in insulin resistance were related to improvements in cognitive function. In another study, the improvements of global cognitive function were explained in part by decreases in peripheral insulin resistance (HOMA-IR) in individuals who participated in more vigorous activities [36]. It is known that hyperinsulinemia is neurotoxic [71], and thus it is possible that insulin sensitivity and insulin signaling pathway improvements after exercise would favor neurogenesis, and thereby improved cognitive function. However, additional animal and human studies are required to determine the nature of the mechanistic links suggested by the relationships reported in this review.

The reductions in HbA1c and the reduction in fasting blood sugar were related to cognitive benefits in one NRCT study. [39] With the limited data available, it is difficult to conclusively determine the specific role of changes in glucose homeostasis on cognition. However, this study does suggest that improvements in glucose homeostasis are potentially related to improvements in memory and executive function of older adults with T2D.

### Limitations of this review and future research

This review was limited by the number of RCTs available in this cohort. Cognitive impairment also introduces the problematic issue that potentially different etiological processes and subtypes may have been mixed within diabetes, insulin resistance or hyperglycemia cohorts included. Several methodological issues were noted in this emerging field. Negligible or nonsignificant effects were found in a high proportion of cognitive outcomes. For all outcomes, inadequate reporting of mean and SD and group mean difference precluded calculation of ES in many studies. Given our overall finding was for no significant effect, the unavoidable issue of negative publication bias should not have impinged upon the outcome of this review. Furthermore, the variety of different exercises modalities (intensity, volume, etc.) might be a limitation. Previous reviews have suggested that aerobic exercise and resistance training should form part of any lifestyle intervention aimed at improving the cognitive function and metabolic profile in healthy adults [16, 72]. However, less of the focus has been on cognitive adaptations for cognitively normal older adults with type 2 diabetes, insulin resistance, or glucose intolerance who are at increased risk of cognitive deficit. This is the first review to focus on neurocognitive adaptations in response to exercise in older adults with T2D, IR, or IGT, as well as attempt to identify any mechanistic links between changes in cognition and changes in metabolism, body composition, neurotrophic factors, and inflammation. In addition, this review and analysis were conducted only among persons using glucose-lowering drugs and/or insulin, as those are the published studies in diabetes with cognitive outcomes. Thus, we cannot clearly answer the question as to whether the relationship between exercise and cognition we observed would also be present in T2D treated only with lifestyle rather than medications for diabetes. The studies included did not systematically report all medication usage in participants, nor did they report subgroup analyses of cognitive adaptations in people on different medication regimens, such as those on insulin vs. oral hypoglycemics for example. Therefore, it was not possible to assess the influence of particular medications on cognitive responses to exercise. Future studies should provide more detail in this regard, and directly compare those on relevant medication regimens.

With only three RCT studies meeting the criteria for this review to date, further research is required to adequately address the relationship between the improvements in metabolic health and improvements in cognition in individuals with T2D. In particular, the data on cognitive function presented in these trials were secondary outcomes for all studies, and as such, the studies may have been underpowered, and there was not much discussion or exploration of the mechanistic links between the exercise and cognitive change. Many other putative mechanistic factors remain to be studied in this regard as well. Additionally, little is known regarding the persistence of therapeutic effects on cognition following the termination of exercise. The training regimes were relatively short and some lacked sufficient intensity to optimize neurophysiological or neuropsychological change. Dose-response relationships between exercise dose (both volume and intensity) and comparisons of different exercise modalities and cognitive outcomes in this cohort are completely lacking, and should also be a focus of the additional investigation. Furthermore, future studies will also need to use cognitive measures which are comprehensive and more sensitive to longitudinal change, and provide long-term follow up to assess the sustainability of any gains achieved during clinical trials. Arguably the most salient issue for the field is the expansion of outcomes to assess transfer of cognitive gains to activities of daily living, quality of life, and psychological well-being.

## Conclusions

The limited data available suggest that aerobic exercise or lifestyle interventions may improve some aspects of cognition in older adults with T2D or IGT, including executive function, delayed memory, and global cognitive scores, but the effects are inconsistent and require further study. In the present review, exercise-induced improvements in insulin sensitivity and glucose levels were associated with the observed cognitive benefits, while there is insufficient evidence exploring any relationship with other physiological adaptations at this time. In addition, literature from other cohorts supports potential advantages for moderate-to-high intensity aerobic exercise, rather than the low-intensity aerobic training paradigms sometimes recommended clinically in T2D or IGT, but confirms the need for dose-response trials in this cohort. Furthermore, comparing study effects was difficult due to different neuropsychological tests used, and standardized cognitive test batteries would increase the ability to synthesize data across trials. Future research in this field should include high quality, robust, randomized controlled trials which enroll older adults with T2D or metabolic syndrome to confirm any beneficial effects of physical exercise on cognition in these cohorts specifically. Moreover, analyses of the relationships between exercise-induced improvements in insulin sensitivity, glucose levels, body composition, and other metabolic parameters that potentially mediate and/or moderate the cognitive adaptations are needed. Such investigations will lead to a better understanding of the underlying mechanisms and therefore to the design of interventions specifically targeting the specific needs of those with metabolic disease. This will promote optimal physical, cognitive and psychological health, and ultimately improve quality of life in such individuals.

## Abbreviations

AD: Alzheimer’s disease
AOS: Anthony J O’Sullivan
BDNF: Brain-derived neurotropic factor
BMI: Body mass index
CI: Confidence intervals
ES: Effect size
HbA1c: Glycosylated hemoglobin
HOMA: Homeostatic Model of Assessment
IGF-1: Insulin-like growth factor-1
IGT: Impaired glucose tolerance
IR: Insulin resistance
JOBA: Intensity horseback riding therapeutic equipment
METs: Metabolic equivalents
MFS: Maria Fiatarone Singh
MMSE: Mini-Mental State Exam
NRCTs: Non-randomized controlled trials
OGTT: Oral glucose tolerance test
PEDro: Physiotherapy Evidence Database scale
PRISMA: Preferred reporting items for systematic reviews
PRT: Progressive resistance training
RCTs: Randomized controlled trials
RRZ: Ren Ru Zhao
SDs: Standard deviations
SLUMS: Saint Louis University Mental Status
T2D: Type 2 diabetes
TMTB: Trail Making Test B
VO_2peak_: Peak oxygen uptake
VS: Versus
